# Priming Exercises and Their Potential Impact on Speed and Power Performance: A Narrative Review

**DOI:** 10.5114/jhk/204371

**Published:** 2025-06-25

**Authors:** Lucas A. Pereira, Piotr Zmijewski, Artur Golas, Krzysztof Kotula, Michael R. McGuigan, Irineu Loturco

**Affiliations:** 1 NAR—Nucleus of High Performance in Sport, São Paulo, Brazil.; 2 Department of Human Movement Sciences, Federal University of São Paulo, São Paulo, Brazil.; 3 Department of Biomedical Sciences, Józef Piłsudski University of Physical Education in Warsaw, Warsaw, Poland.; 4 Institute of Sport Sciences, The Jerzy Kukuczka Academy of Physical Education in Katowice, Katowice, Poland.; 5 Sports Performance Research Institute New Zealand (SPRINZ), Auckland University of Technology, Auckland, New Zealand.; 6 UCAM Research Center for High Performance Sport, UCAM Universidad Católica de Murcia, Murcia, Spain.

**Keywords:** resistance training, athletes, muscle strength, athletic performance, sprint velocity

## Abstract

Improving the competitive level of elite athletes is always a considerable challenge for coaches, regardless of the sport discipline or a training phase. From this perspective, researchers and sport scientists continuously seek more effective training methods, capable of inducing acute changes or long-term adaptations in athletes with diverse training backgrounds. Recently, priming exercises have emerged as a promising approach to enhance athletic performance over short periods of time, either before sport-specific training sessions or official competitions. By incorporating different priming protocols into their regular practices—including sets of traditional resistance exercises, ballistic exercises or sprint drills—athletes have consistently achieved significant improvements in their speed and power qualities within time intervals ranging from 2 h to 48 h. This narrative review summarizes and examines the main studies on this topic, while providing practitioners with theoretical perspectives, practical insights, and guidelines for implementing efficient priming protocols in their training routines. In conclusion, priming interventions generally produce positive outcomes, irrespective of the exercise type and athletes’ backgrounds, and may facilitate the transfer of these benefits to certain sport-specific tasks.

## Introduction

Elite athletes are known for their exceptional physical and competitive performance (Cormie et al., 2011; Loturco et al., 2023a; Mckay et al., 2022). In this context, practitioners are constantly seeking more effective training strategies to enhance their broad spectrum of physical abilities. However, recent studies have revealed only slight—or even non-existent—variations in the physical qualities of elite athletes throughout the annual training season (Freitas et al., 2021; Gabbett, 2005; Loturco et al., 2024a; Pecci et al., 2023), specifically in strength-, speed-, and power-related abilities. Somewhat surprisingly, this phenomenon appears to be independent of the training method, the season period, and even the athletes’ competitive level (e.g., regional or world-class sprinters and professional rugby players) (Freitas et al., 2021; Gabbett, 2005; Loturco et al., 2023a; Pecci et al., 2023). Given this, the use of pre-conditioning strategies has been raised as an important alternative approach to acutely improve performance, particularly in the phases leading up to competitions.

In this regard, various protocols of post-activation performance enhancement (PAPE) (Boullosa, 2021; Garbisu-Hualde and Santos- Concejero, 2021; Seitz and Haff, 2016) and priming exercises (e.g., short-duration, high-intensity exercises performed 2 h to 48 h before a competition or the key training session to enhance physical performance) (Harrison et al., 2019; Mason et al., 2020; Valcarce-Merayo and Latella, 2023) have been utilized by athletes across different sports and competitive levels (Harrison et al., 2020). Although PAPE and priming exercises can be applied for the same purpose and may be regarded as similar stimuli, important differences exist between these methods and their respective procedures (Valcarce-Merayo and Latella, 2023). While PAPE can serve as a complimentary task implemented during traditional warm-up routines to acutely improve subsequent performance for 3 min to 10 min (Boullosa, 2021), priming exercises are commonly prescribed over a longer period before the target activity (e.g., up to 48 h before a competition) (Harrison et al., 2019, 2020). Importantly, although PAPE is a well-established strategy (Boullosa, 2021; Garbisu-Hualde and Santos-Concejero, 2021; Loturco et al., 2024b), some uncertainty persists regarding its effectiveness, which may be even more critical in high-performance environments (Garbisu-Hualde and Santos-Concejero, 2021; Seitz and Haff, 2016). For example, a recent meta-analysis found no significant PAPE effects on the sprint performance of competitive sprinters after analyzing the acute responses to distinct conditioning activities (CAs) (Loturco et al., 2024b). Additionally, studies that demonstrated the effectiveness of PAPE approaches revealed a very limited application in real sport settings, as these methods often require multiple sets of high-intensity exercises (i.e., squats performed with loads exceeding 80% of one-repetition maximum [1RM]) (Garbisu-Hualde and Santos-Concejero, 2021; Seitz and Haff, 2016). Therefore, practitioners might be reluctant to incorporate this strategy into warm-up routines prior to actual competitions.

In contrast, the less studied concept of priming activities (e.g., also named “delayed potentiation”) has shown promising and consistent results, specifically in increasing speed and power production, when applied to athletes from diverse sports and training backgrounds (Harrison et al., 2019; Mason et al., 2020). A previous survey study revealed that among 69 practitioners, 51% regularly prescribed priming exercises, and 84% believed that resistance-based priming sessions were beneficial for physical and technical performance (Harrison et al., 2020). From a practical standpoint, priming exercises are easier and safer to implement during pre-competitive periods, compared to PAPE strategies, as positive effects have been observed following the application of light-loaded ballistic exercises performed up to 24 h pre-competition (Loturco et al., 2025b, 2025c). In elite sports, characterized by congested schedules, restricted time for strength, speed, and power training, and logistical constraints related to long journeys and complex recovery methods, these findings are especially relevant. Unlike certain PAPE strategies used with highly trained subjects (Garbisu-Hualde and Santos-Concejero, 2021; Seitz and Haff, 2016), priming sets do not necessarily require heavy loads and can be implemented even in facilities with limited infrastructure and equipment, such as hotel gyms. Additionally, light-loaded exercises induce low levels of perceived exertion (Loturco et al., 2024c, 2025c) and, consequently, minimal residual fatigue, improving the confidence of practitioners in applying this strategy close to competitions.

Given the importance of priming strategies and their potential application in real-world training contexts, it is essential to critically analyze and discuss the actual effectiveness of this contemporary training approach in elite athletes. The current narrative review aimed to provide a comprehensive overview and examine the findings of previous research that investigated the effects of priming protocols on the strength, speed, and power abilities of athletes across different sports. To align with this objective, this review included studies that met the following criteria: 1) athletes from various sports, regardless of the competitive level and the training background; 2) studies implementing CAs with a time interval between the priming exercise and the target measurement ranging from at least 2 h to a maximum of 48 h; and 3) resistance-based priming exercises, including traditional and ballistic lower- and upper-body exercises, as well as plyometric and sprint drills. This narrative review was structured into distinct sub-sections to provide a detailed examination of the physiological and neuromuscular mechanisms underlying priming responses, the multiple protocols utilized, the optimal time window for achieving the most pronounced effects, and practical considerations for implementing this strategy prior to competition or sport-specific training sessions.

## Physiological Mechanisms Related to Priming Effects

The physiological mechanisms primarily associated with or capable of explaining the positive effects of priming exercises are not yet fully understood. In fact, most of the hypotheses proposed in this regard have emerged from studies involving PAPE and chronic training interventions (Boullosa, 2021; Harrison et al., 2019; Mason et al., 2020; Pratt, 2023). For example, it is expected that increases in muscle temperature, stiffness, and sensitivity to calcium ions—factors commonly associated with PAPE responses—are also expected to contribute to priming responses (Boullosa, 2021; Harrison et al., 2019; Mason et al., 2020; Pratt, 2023). Similarly, following resistance training interventions, increases in motor unit firing frequency, recruitment, and synchronization are consistently observed and could also be relevant in the context of delayed potentiation (Boullosa, 2021; Harrison et al., 2019; Mason et al., 2020; Pratt, 2023). Previous research has examined neuromuscular responses after applying priming exercise protocols, using indirect markers of neural changes to elucidate the underlying mechanisms of this approach (Donghi et al., 2021; Pratt, 2023; Tsoukos et al., 2018). In this sense, Donghi et al. (2021) and Pratt (2023) investigated the effects of priming sessions on the percentage change in voluntary activation—an indirect measure of motor unit recruitment—and on the rate of torque development (RTD), which is indirectly related to motor unit recruitment, discharge rates, and stiffness. Pratt (2023) observed significant improvements in voluntary activation and RTD compared to a control condition 6 h after prescribing a priming session consisting of 2 sets of 2 repetitions of half squats (HSs) performed at 87% of 1RM for 16 physically active men. In contrast, Donghi et al. (2021) did not detect any meaningful changes in either voluntary activation or RTD 5.5 h after applying two distinct priming protocols: one involving repeated sprint running and the other combining HSs, ladder drills, and 20-m sprints in young soccer players. Additionally, Tsoukos et al. (2018) showed significant increases in the rate of force development (a variable similar to the RTD) in 17 well-trained power and team-sport athletes 24 h after completing a short training session incorporating 5 sets of 4 jump squats (JSs) at 40% of 1RM. These results suggest that priming strategies can induce substantial changes in certain neuromuscular capabilities, particularly when resistance training sessions with a relatively low volume of strength-power exercises are implemented. However, due to the limited number of studies assessing multiple variables related to neuromuscular function, further research on this topic is clearly needed (Donghi et al., 2021; Pratt, 2023; Tsoukos et al., 2018).

Variations in hormonal responses are another potential factor regularly associated with priming effects, especially fluctuations in testosterone and cortisol levels. These hormones are often linked to athletic performance and are frequently measured to evaluate changes (i.e., increases or decreases) following different priming protocols (Cook et al., 2014; Donghi et al., 2021; Harrison et al., 2021; Russell et al., 2016). For example, Cook et al. (2014) analyzed the effects of two priming activities on salivary testosterone levels in semi-professional rugby players: one involving 5 x 40-m sprints and another comprising 12 repetitions of bench press and squat exercises in an incremental testing protocol. That study revealed that priming exercises attenuated the diurnal decline in testosterone concentration (i.e., contrasting morning vs. afternoon periods) compared to a control condition. However, baseline hormonal levels were higher in the priming protocol compared to the control condition. Russell et al. (2016) reported a similar effect in professional rugby players after completing either 5 sets of the bench press at 75% 1RM or 6 x 40-m sprints with a 180^o^ directional change. The diurnal reductions in salivary testosterone levels were less pronounced after the priming sessions compared to the control condition. Moreover, in the study by Donghi et al. (2021), the within-day decrease in blood testosterone was “possibly” attenuated (i.e., analyzed via magnitude-based inferences method) in the repeated sprint priming session compared to the control, whereas the priming protocol did not alter cortisol responses relative to the control condition. Finally, Harrison et al. (2021) did not observe priming effects from back squats executed under lighter (65% 1RM) or heavier (> 67% 1RM) loading conditions on salivary testosterone and cortisol concentrations when compared to the control. Considering that testosterone levels may be affected by the circadian rhythm, these effects tend to be more pronounced for a few hours after completing the priming protocol (e.g., up to 6 h). However, it remains unknown how these hormonal variations could influence priming over longer time periods (e.g., ≥ 24 h) (Harrison et al., 2019).

## Priming Exercise Protocols

**Figure 1:**
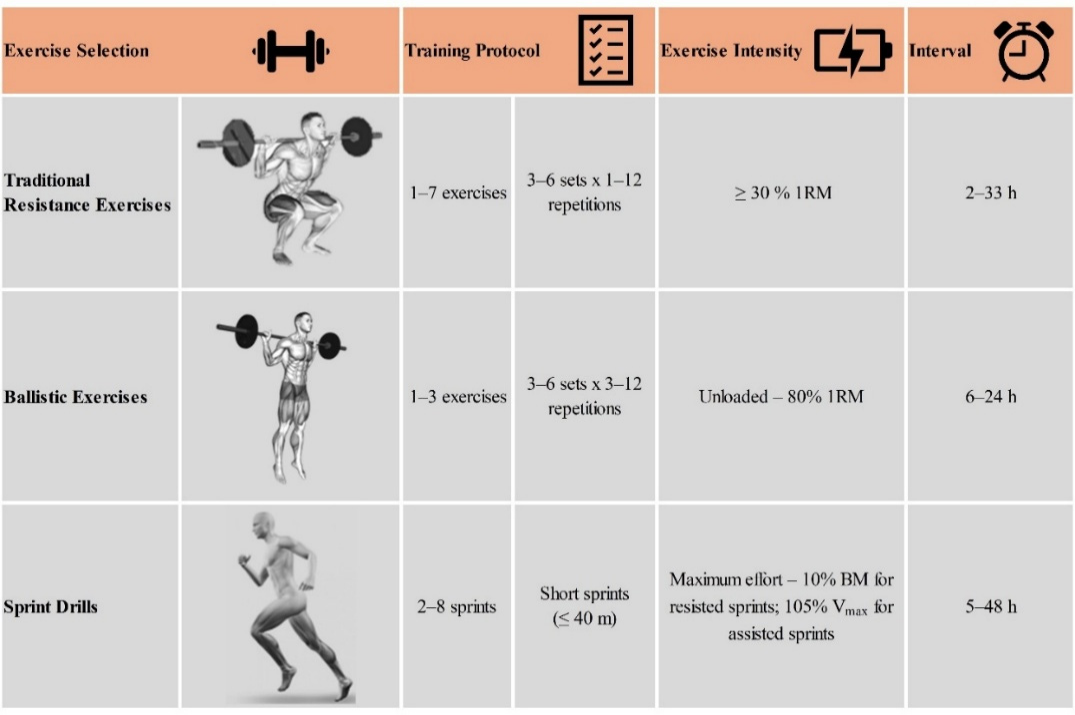
Overview of average data on exercise types, training protocols, exercise intensity, and the interval between priming interventions and performance tests across the analyzed studies.

Multiple priming exercise protocols have been implemented in subjects with different training backgrounds (Harrison et al., 2019; Mason et al., 2020; Valcarce-Merayo and Latella, 2023). In the following sections, we present the effects of various resistance priming sessions on the neuromuscular abilities of athletes from different sports. To facilitate the understanding of priming effects, the sections are divided into protocols involving traditional and ballistic resistance exercises, as well as maximal sprinting speed drills. The time between the priming session and the target activity ranges from 2 h to 48 h (Mason et al., 2017; Tsoukos et al., 2018). Figure 1 provides a general presentation of the priming protocols used in the different studies testing traditional and ballistic exercises, and sprint drills.

## Traditional Resistance Exercises

Due to their popularity and effectiveness in improving physical performance both acutely (i.e., immediate effects) and chronically (i.e., after training interventions), traditional exercises are frequently employed by practitioners during resistance training sessions and in studies examining priming effects (Harrison et al., 2019; Loturco et al., 2024b, 2024d). The most commonly tested exercises are the squat and its variations (e.g., back squats and HSs), performed with varying loading intensities, sets, and repetitions. In general, priming sessions involving squat exercises with low volume (e.g., ≤ 6 sets of ≤ 4 repetitions) and heavy loads (≥ 80% 1RM) (Cook et al., 2014; Raastad and Hallén, 2000; Saez De Villarreal et al., 2007; Wang et al., 2024) or progressive loading strategies covering a wide range of loads (i.e., from 30% to 95% 1RM) (Cook et al., 2014) (Table 1) have been shown to significantly improve sprint and power performance in competitive athletes (Cook et al., 2014; Saez De Villarreal et al., 2007; Wang et al., 2024).

**Table 1:** Summary of studies on the effects of priming sessions with traditional resistance exercises.

Reference	Subjects	Priming protocol	Interval	Outcomes
Cook et al., 2014	18 semi-professional rugby union players	BP and Squat 3 × 50% (of 3RM), 3 × 80%, 3 × 90%, and 3 × 100%	6 h	↑ 3RM BP; ↑ 3RM squat; ↑ 40-m sprint performance; ↑ CMJ PPO
Donghi et al., 2021	12 young soccer players	Control	≈ 5.5 h	*^#^possibly* ↑ CMJ height; *possibly* ↑ 20-m + 20-m sprint performance
		4 x 12 HS, 6 speed ladder drills, 20-m sprints		*possibly* ↑ CMJ height; *likely* ↑ 20-m + 20-m sprint performance
Ekstrand et al., 2013	14 throwers	Back squat performed to failure and power clean	4-6 h	↑ backward overhead shot throw; ↔ VJ PP
Mason et al., 2017	13 state-level rugby players	Control	2 h	↔ PV, PF, PP in 20-kg bench throw and CMJ
		4 x 3 banded back squat; 4 x 3 banded bench press		↔ PV, PF, PP in 20-kg bench throw and CMJ
Nutt et al., 2022	16 cricketers	1 x 4 trap bar deadlift at 50%, 70%, and 80% RM; 3 x 4 at 85% RM	5.5 h	↑ CMJ height; ↑ ‘run-two’ test (35.36-m sprint)
Raastad and Hallén, 2000	8 powerlifters, 1 javelin-thrower and 1 speed-skater,	3 x 3 RM squat and front squat and 3 x 6 RM knee extension	3-33 h	↓ SJ height
		3 x 3 (70% 3 RM) squat and front squat and 3 x 6 (70% 6 RM) knee extension		↑ SJ height
Russell et al., 2016	15 professional rugby players	5 x 10 bench press at 75% RM	5 h	↑ RSA best time; ↔ CMJ height and RSA total time
Saez de Villarreal et al., 2007	12 trained volleyball players	Control	6 h	↔ CMJ height, DJ height, and CMJ height with optimal load
		2 x 4 reps at 80% 1RM HS; 2 x 3 at 85% 1RM HS		↑ DJ height; ↔ CMJ height, and CMJ height with optimal load
		2 x 4 reps at 80% 1RM HS; 2 x 2 at 90% 1RM HS; 2 x 1 at 95% 1RM HS		↑ DJ height; ↔ CMJ height and CMJ height with optimal load
		3 x 5 reps at 30% 1RM HS		↔ CMJ height, DJ height, and CMJ height with optimal load
Wang et al., 2024	16 male highly trained national level collegiate athletes (basketball, volleyball, track and field)	3 x 3 back squats at 85% RM (no rest between repetitions; TS)	6 h	↑ CMJ height, 20-m sprint and T-Test performance
		3 x 3 back squat at 85% RM (30-s interval between repetitions; CS)		↑ CMJ height, 20-m sprint and T-Test performance. *Higher increases than under the TS condition
Woolstenhulme et al., 2004	18 division I women basketball players	7 exercises performed in 3–6 sets at loads ranging from a 5 to 12 RM	6 h	↔ CMJ height

Note: BP: bench press; RM: repetition maximum; HS: half squat; TS: traditional set; CS: cluster set; CMJ: countermovement jump; PPO: peak power output; VJ: vertical jump: PP: peak power; PV: peak velocity; PF: peak force; SJ: squat jump; RSA: repeated sprint ability test; DJ: drop jump. ↑ significant increase; ↔ no significant change; ↓ significant decrease; ^#^analyzed via magnitude-based inference method

Wang et al. (2024) compared the effects of two distinct priming sessions involving 3 sets of 3 repetitions of the back squat performed at 85% 1RM, with or without a 30-s rest interval between repetitions, in highly trained male national-level collegiate athletes (i.e., basketball, volleyball, track and field athletes). Significant increases in countermovement jump (CMJ) height, 20-m sprinting speed, and T-Test time were observed 6 h after the priming session, with greater improvements under the cluster condition (e.g., with a 30-s rest interval). Nutt et al. (2022) reported positive effects on CMJ and “run-two test” (i.e., 35.36-m sprint) performance in cricketers 5.5 h after a priming session. The protocol involved 6 sets of deadlifts executed in a progressive loading manner, ranging from 50% to 85% 1RM. Furthermore, Saez De Villarreal et al. (2007) examined various protocols involving HSs at 80% and 85% 1RM, as well as a progressive loading scheme at 80%, 90%, and 95% 1RM, in trained volleyball players. Significant and comparable improvements in drop jump (DJ) height were detected 6 h after both types of priming sessions.

In contrast, in the same study by Saez De Villarreal et al. (2007), a priming session with lighter loads (30% 1RM) did not elicit any significant positive or negative effects. However, it is worth noting that traditional exercises performed under very light loading conditions usually exhibit a very aggressive braking phase at the end of the concentric portion (Cormie et al., 2011; Loturco et al., 2023c, 2025a), which may compromise the potential positive (priming) effects—a mechanical aspect that will be better explored in the topic below—of ballistic exercises. Similarly, Mason et al. (2017) found no priming effects 2 h after a resistance training session that included “banded back squats” (e.g., an additional 46.8 kg of tension applied to an Olympic barbell) and bench press exercise in state-level rugby players. In a study of Division I collegiate women’s basketball players, Woolstenhulme et al. (2004) did not observe significant effects on CMJ performance 6 h after a priming session consisting of multiple traditional exercises (i.e., Olympic weightlifting derivatives, back squats, and bench presses) performed in 3 or 4 sets with loads ranging from 5 RM to 12 RM.

Two studies have examined delayed potentiation effects using upper body exercises as priming strategies (Cook et al., 2014; Russell et al., 2016). Cook et al. (2014) investigated the effects of 4 sets of 3 repetitions of bench press and squat exercises with loads ranging from 50% to 100% 3RM (i.e., using either half of the load or the total load that allowed the completion of 3 RM) in semi-professional rugby union players. Significant improvements in CMJ peak power, 40-m sprint performance, and 3RM strength in both bench press and squat exercises were detected 6 h after the priming session. Finally, Russell et al. (2016) demonstrated a positive effect in the first two sprints (*p* = 0.003 and *p* = 0.01 for the first and second sprint, respectively) of a repeated sprint test in professional rugby players 5 h after completing a priming session that included 5 sets of 10 repetitions of the bench press at 75% 1RM. Interestingly, those authors suggested that the effect of an upper-body exercise on sprint performance might be attributed to changes in hormonal responses induced by the resistance training session when compared to a control condition.

A summary of studies analyzing the effects of priming sessions using traditional resistance exercises is presented in Table 1. In general, when incorporating resistance exercises into priming strategies, short sessions comprising one or two exercises performed under moderate-to-heavy loading conditions (i.e., from 50% to ≈85% 1RM) with a low volume of sets and repetitions are recommended. Moreover, the time interval between the priming session and the performance test ranged from 2 h to 6 h, with the greatest effects observed for intervals of 4 h and 6 h.

## Ballistic Exercises

Ballistic exercises involve movements in which the body (e.g., unloaded or loaded jumps) or an implement (e.g., a medicine ball) is projected, irrespective of the movement direction (i.e., vertical or horizontal). The primary mechanical characteristic of these exercises is that, during ballistic movements, individuals accelerate their bodies (or the implement) and apply force continuously throughout the entire concentric phase until the point of the projection (or take-off) (Cormie et al., 2011; Loturco et al., 2023c, 2024d; Sanchez-Medina et al., 2010). This specific attribute (i.e., a movement that is entirely accelerative) contrasts with traditional exercises (e.g., a HS), particularly under light-load conditions, where athletes must apply a force opposing the movement (i.e., during the braking phase) to decelerate and stop the lift at the end of the concentric phase (Loturco et al., 2023c; Sanchez-Medina et al., 2010). These distinct mechanical differences provide a significant advantage to ballistic exercises compared to traditional exercises, as continuous acceleration is a fundamental requirement in most sport-specific tasks, such as jumping, sprinting, kicking, and punching (Cormie et al., 2011; Loturco et al., 2021, 2023). For these reasons, ballistic exercises are frequently reported as one of the most commonly prescribed exercises for elite athletes across various sport disciplines (Cormie et al., 2011; Loturco et al., 2023d, 2024d, 2025c). Indeed, several studies consistently confirm the effectiveness of ballistic exercises (e.g., loaded and unloaded JSs) in enhancing multiple physical qualities (e.g., vertical jump performance, sprinting ability, and maximum strength) in elite athletes (Cormie et al., 2011; Loturco et al., 2024d, 2025c).

Regarding their use as priming activities, ballistic exercises have been widely implemented with team-sport athletes (Loturco et al., 2024d, 2025b, 2025c), whereas only one study has examined their effects in well-trained swimmers (Zaras et al., 2022). In general, the protocols included no more than 6 sets per exercise and a maximum of 3 exercises per session (Table 2). For example, Loturco et al. (2025c) demonstrated similarly significant effects of two priming exercise protocols in which elite female rugby seven players performed 6 sets of 6 JSs at 40% or 80% HS 1RM. CMJ height and 40-m linear sprint speed improved after 6 h, while the change of direction speed increased both 6 and 24 h post-priming protocol under both conditions. Importantly, after the lighter loading condition (i.e., 40% 1RM), players reported lower levels of perceived exertion compared to the heavier loading condition (i.e., 80% 1RM), which may be considered a highly relevant aspect in high-performance sports, where fatigue is a constant factor (Loturco et al., 2024c, 2025c). Tsoukos et al. (2018) demonstrated a positive effect 24 h after performing 5 sets of 4 JSs executed at 40% HS 1RM on CMJ and DJ performance in well-trained male power and team-sport athletes. Notably, the improvements in CMJ height persisted up to 48 h. In addition, Saez De Villarreal et al. (2007) observed positive priming effects 6 h after completing a protocol consisting of 3 sets of 5 CMJs performed under an optimal loading condition (i.e., the load that maximized power output). However, no significant effects were observed following 3 sets of 5 DJs executed from the optimal drop height (i.e., the height associated with the best reactive strength index). Similarly, Loturco et al. (2025b) reported more pronounced priming effects on CMJ height and 30-m sprint speed at 6 h, as well as on 10- and 30-m sprint speed and JS bar velocity at 30% and 100% of body mass (BM) 24 h after a ballistic session comprising 6 sets of 6 JSs at 40% HS 1RM in male rugby union players. Conversely, in another protocol involving 6 sets of 6 DJs from the optimal height, improvements were only detected in JS bar velocity for both 30% and 100% BM at 24 h (Loturco et al., 2025b). Lastly, Panteli et al. (2024) identified priming effects in T-Test performance, but not in CMJ height and 10- and 20-m sprint speed 24 h after a session including JSs and jump shrugs with a 15-kg barbell and DJs performed from a 30-cm box height.

**Table 2:** Summary of studies on the effects of priming sessions with ballistic exercises.

Reference	Subjects	Priming protocol	Interval	Outcomes
Loturco et al., 2025b	20 male rugby union players	6 x 6 JS at 40% RM	6 h and 24 h	↑ CMJ height and 30-m sprint performance at 6 h; ↑ 10- and 30-m sprint performance, and JS PV at 24 h
		6 x 6 DJ best RSI		↑ JS PV at 24 h
Loturco et al., 2025c	20 female rugby seven players	6 x 6 JS at 40% RM	6 h and 24 h	↑ CMJ height, 40-m sprint, and COD performance at 6 h; ↑ COD performance and JS PV at 24 h
		6 x 6 JS at 80% RM		↑ CMJ height, 40-m sprint, and COD performance at 6 h; ↑ COD performance at 24 h
Panteli et al., 2023	14 well-trained soccer players	Ballistic power: 4 x 8 JS; 4 x 8 jump shrugs; 3 x 6 DJ 30-cm	24 h	↑ T-Test performance; ↔ CMJ, 10- and 20-m sprint.
Saez de Villarreal et al., 2007	12 trained volleyball players	3 x 5 jumps with optimal loaded CMJ	6 h	↑ DJ height and CMJ height with optimal load; ↔ CMJ height
		3 x 5 DJ from optimal height		↔ CMJ height, DJ height, and CMJ height with optimal load
Tsoukos et al., 2018	17 well-trained male power and team sport athletes	5 x 4 JS at 40% RM	24 h and 48 h	↑ CMJ height at 24 h and 48 h; ↑ DJ RSI and RFD at 24 h; ↔ LP MIF at 24 h and 48 h; ↔ DJ RSI and RFD at 48 h;
Zaras et al., 2022	13 well-trained adolescent swimmers	3 x 8 slam balls; 3 x 8 CMJ; 3 x 12 stretch cords upper body swimming	24 h	↑ 50-m crawl performance; ↔ CMJ height

Note: JS: jump squat; RM: repetition maximum; DJ: drop jump; RSI: reactive strength index; CMJ: countermovement jump; COD: change of direction; PV: peak velocity; RFD: rate of force development; LP: leg press; MIF: maximum isometric force; ↑ significant increase; ↔ no significant change

The aforementioned results reinforce the effectiveness of ballistic exercises, not only as performance-enhancing strategies, but also as a form of priming exercise for improving the neuromuscular performance of competitive athletes. A summary of studies analyzing the effects of priming protocols using ballistic exercises is presented in Table 2. By examining the table, it becomes evident that priming sessions consisting of multiple sets of ballistic exercises with light loads (i.e., ≤ 40% HS 1RM) are highly effective in optimizing athletic and sport-specific performance, while minimizing acute (and potentially accumulated) fatigue, which is typically induced by heavy loading protocols (i.e., ≥ 80% 1RM) (Loturco et al., 2024c, 2025c). In contrast, priming sessions involving unloaded jumps did not result in meaningful performance improvements. This suggests that the mechanical stimuli provided by such exercises may be insufficient to elicit significant changes in strength, speed, and power performance. Further research may be warranted to investigate priming protocols incorporating multiple types of unloaded and demanding jumps, such as hurdle jumps or multiple bounding, to induce greater neuromuscular responses. Finally, it is worth noting that in all studies, the time interval between priming sessions and performance tests ranged from 6 h to 48 h, with the most pronounced effects observed at the 6^th^ h and the 24^th^ h. Coaches should take these guidelines into account when designing their priming training strategies.

## Maximal Sprints

The utilization of maximal sprints is a common strategy in studies investigating the effects of priming interventions on competitive athletes. Notably, maximal-speed sprinting is the primary method employed by Olympic sprint and jump coaches for speed development, who also incorporate maximal sprint drills into their warm-up routines (Kotuła et al., 2023; Loturco et al., 2023b, 2024b). Moreover, resisted and assisted sprints are widely used methods among practitioners from different sports in this context (i.e., as CAs, during warm-up routines). In addition to their widespread use, maximal sprint efforts have been shown to elicit positive performance responses when incorporated into priming protocols (Cook et al., 2014; Kotuła et al., 2023; Loturco et al., 2024b; Pino-Mulero et al., 2025).

Priming protocols based on maximal sprint efforts typically involve short sprint distances and cycle ergometer sprints (i.e., ≈6-s or ≤ 40-m) performed under unresisted conditions or with the use of assisted or resisted sprints. For example, Cook et al. (2014) demonstrated that a priming session consisting of 5 x 40-m sprints in semi-professional rugby union players improved 40-m sprint performance 6 h after the protocol. Similarly, Kotuła et al. (2023) examined the effects of three distinct priming sessions in female sprinters: 1) 4 x 40-m resisted sprints with a load equivalent to 10% BM, 2) 4 x 40-m assisted sprints at 105% of maximum sprint speed, and 3) a combined sprint session comprising 2 x 40-m sprints for each assisted and resisted sprint condition. As a result, 30- and 50-m sprint speed increased significantly 48 h after the assisted and combined priming sessions but not following the resisted condition. Pino-Mulero et al. (2025) observed significant improvements in CMJ height and 20-m sprint performance 24 h after a priming session in which under-18 semi-professional soccer players performed two sets of 2 x 15-m sled pushes with a load equivalent to 100% BM. Finally, Russell et al. (2016) reported positive performance enhancements in CMJ height 5 h after a priming session consisting of 6 sets of 6-s cycle ergometer sprints with a resistance of 7.5% BM in professional rugby players. Additionally, in the same study, enhancements in CMJ height and the best time of a repeated sprint test were observed 5 h after a session comprising 6 sets of 40-m sprints with a 180^o^ directional change at the midway point (i.e., 20-m).

Although priming studies involving maximal sprints are less numerous than those focusing on other approaches (e.g., traditional resistance and ballistic exercises), the findings to date support the use of the different sprint forms (i.e., unresisted, assisted, and resisted sprints) as effective priming strategies. However, the marked differences among the sprinting protocols preclude more robust conclusions regarding these strategies. Table 3 presents a summary of the priming interventions utilizing sprint drills. Based on a general overview, sprint-based priming exercises should involve no more than 6 sets of short sprints (e.g., ≤ 40-m). When using resisted or assisted sprints, light loading conditions that minimize disruptions in sprinting technique (Loturco et al., 2023b; Zabaloy et al., 2023) —whether by reducing or increasing sprint speed (i.e., 10% BM for resisted sprints or 105% of maximum sprinting speed for assisted sprints)—may be preferred. The adherence to these variables can help practitioners across various sports in selecting the most effective positive (resisted sprints) or negative (assisted sprints) loading ranges for their athletes.

**Table 3:** Summary of studies on the effects of priming sessions with sprint drills.

Reference	Subjects	Priming protocol	Interval	Outcomes
Cook et al., 2014	18 semi-professional rugby union players	5 x 40-m sprints	6 h	↑ 40-m sprint performance
Donghi et al., 2021	12 young soccer players	Control	≈5.5 h	*^#^possibly* ↑ CMJ height; *possibly* ↑ 20 m + 20-m sprint performance
		Repeated sprint running: 6 x 40 m		*possibly* ↑ 20 m + 20-m sprint
Kotula et al., 2023	10 female sprinters	RST: 4 x 40 m with 10% BM	48 h	↓ 20- and 30-m sprint performance; ↔ 50-m sprint performance
		AST: 4 x 40 m with 105% V_max_		↔ 20-m sprint performance; ↑ 30- and 50-m sprint performance
		COMB: 2 x 40 m with 10% BM; 2 x 40 m with 105% V_max_		↔ 20-m sprint performance; ↑ 30- and 50-m sprint performance
Panteli et al., 2023	14 well-trained soccer players	Repeated sprint: 2 x 4 x 15 m; 2 x 4 DJs (30-cm box height) + 10-m sprint	24 h	↑ T-Test performance; ↔ CMJ, 10- and 20-m sprint
Pino-Mulero et al., 2025	16 young semi-professional football	Control	24 h	↔ CMJ height and 20-m sprint performance
		2 x 2 15-m sled push with 100% BM		↑ CMJ height and 20-m sprint performance
Russell et al., 2016	15 professional rugby players	6 x 6-s cycle ergometer sprints	5 h	↑ CMJ height; ↔ RSA best and total times
		6 x 40-m sprints with 180^o^ COD		↑ CMJ height and RSA best time; ↔ RSA total time
Zaras et al., 2022	13 well-trained adolescent swimmers	4 x 50-m crawl swim starting from blocks	24 h	↑ 50-m crawl performance; ↔ CMJ height

Note: RST: resisted sprint training; AST: assisted sprint training; COMB: combined; BM: body mass; Vmax: maximal velocity; DJ: drop jump; COD: change of direction; CMJ: countermovement jump; RSA: repeated sprint ability test. ↑ significant increase; ↔ no significant change; ↓ significant decrease; ^#^analyzed via magnitude-based inference method

## Practical Implications and Future Directions

The application of priming exercises in real-sport environments is highly recommended for coaches, especially before sport-specific training sessions, matches or competitions. However, determining the most effective priming methods remains challenging due to the high variability of stimuli and approaches used in priming studies. Despite this limitation, priming exercises can be viewed positively, as numerous strategies involving traditional resistance, ballistic, and sprinting exercises have been shown to effectively enhance subsequent speed-power performance. In this context, practitioners can select the most appropriate exercises and methods from the options presented here, based on their experience, personal preferences, and, most importantly, the specific characteristics of their sports and competitions. For example, during away competitions or matches where training facilities may be limited, coaches can implement ballistic exercises with light loads, elastic bands or even short sprint drills as priming stimuli. When adequate facilities are available, traditional strength-power exercises can be utilized within the volume and loading ranges provided in this study. As a general recommendation, priming protocols should include one exercise type (a maximum of two for traditional resistance exercises), with a total volume of 3–4 sets for resistance exercises (e.g., squats), 5–6 sets for ballistics (e.g., jump squats), and 2–6 sprints over distances ≤ 40-m (Table 4). It should be noted that these guidelines are specifically related to improvements in speed-power qualities. The limited number of studies, along with the variability in the methods and exercises used to assess maximum strength, prevents us from drawing more robust conclusions in this regard. Future research should investigate the effectiveness of priming sessions that incorporate multiple and more complex intervention protocols (e.g., combining plyometrics with maximal sprint drills or pairing traditional resistance exercises using moderate to heavy loads with ballistic movements performed at lighter loads) to determine the extent to which these mixed approaches can optimize—or potentially compromise—the physical performance of elite athletes. These studies may also be designed to further elucidate how priming exercises elicit responses such as neuromuscular and metabolic changes, with a particular focus on the types of exercises used to induce these specific effects.

**Table 4:** Most effective priming protocols to improve vertical jump, linear speed, and change of direction performance in competitive athletes (based on the analyzed studies).

Priming Strategy		VJ	Linear sprint	COD
***Traditional resistance exercises***	*Loading condition*			
	Light loads (≤ 40% 1RM)	*	*	*
	Multiple loads	++	+	+
	Heavy loads (≥ 80% 1RM)	++	++	+
	*Number of exercises*			
	1	+++	+++	+++
	2	?	+	*
	≥ 3	*	*	*
	*Time interval*			
	2–6 h	+	++	++
	≥ 24 h	?	*	*
***Ballistic exercises***	*Loading condition*			
	Unloaded (plyometrics)	?	*	*
	Light loads (≤ 40% 1RM)	+++	++	++
	Heavy loads (≥ 80% 1RM)	+	+	+
	*Number of exercises*			
	1	+++	++	++
	2	*	*	*
	≥3	*	?	+
	*Time interval*			
	4–6 h	++	++	++
	≥ 24 h	++	?	+++
***Sprint drills***	*Loading condition*			
	Unresisted	*	+++	?
	Resisted	++	?	*
	Assisted	*	+	*
	*Number of exercises*			
	1	?	++	?
	2	*	?	+
	≥3	*	*	*
	*Time interval*			
	5–6 h	?	++	*
	≥ 24 h	?	++	+

VJ: vertical jump; COD: change of direction; 1RM: one-repetition maximum. “+” level of evidence related to distinct priming strategies, the higher the number, the higher the evidence; “?” means conflicting results; * means lack of evidence
